# Application of Citrus and Apple Fibers for Formulation of Quercetin/Fiber Aggregates: Impact of Quercetin Concentration

**DOI:** 10.3390/plants11243582

**Published:** 2022-12-19

**Authors:** Ivana Buljeta, Ina Ćorković, Anita Pichler, Josip Šimunović, Mirela Kopjar

**Affiliations:** 1 Faculty of Food Technology Osijek, Josip Juraj Strossmayer University of Osijek, Franje Kuhača 18, 31 000 Osijek, Croatia; 2 Department of Food, Bioprocessing and Nutrition Sciences, North Carolina State University, Raleigh, NC 27695, USA

**Keywords:** quercetin, apple fiber, citrus fiber, HPLC, structural changes, thermal stability, antioxidant activity

## Abstract

Among flavonoids, quercetin has gained special attention due to its positive biological activities. Quercetin’s disadvantages, such as its hydrophobic nature, poor solubility, and permeability, could be overcome by complexation with different polymers. Dietary fibers are known as carriers of polyphenols, which can protect them from environmental conditions and thus allow them to be absorbed. In this study, apple and citrus fibers (as applicable food by-products) were used as carriers of quercetin. A constant amount of fibers (1%) and different concentrations of quercetin solution (5 mM, 10 mM, and 20 mM) were complexed. Obtained fiber aggregates were subjected to HPLC to determine the quercetin concentration and antioxidant activity of aggregates (ABTS, DPPH, FRAP, and CUPRAC assays). IR spectra were recorded to confirm complexation of quercetin with selected fibers, and an additional DSC study was performed to evaluate the thermal stability of fiber aggregates. The results of HPLC analysis showed that quercetin had higher affinity towards apple fiber than citrus fiber, without proportional trends of adsorption. Consequently, apple fiber aggregates had higher antioxidant potential than citrus fiber aggregates. FTIR-ATR analysis showed the formation of new bands and the loss of existing bands when quercetin was present. Adsorption of quercetin also had an impact on the thermal stability of formulated fiber aggregates. For apple fiber, this impact was negative, while for citrus fiber, the impact was positive. These results could contribute to greater understanding of quercetin’s behavior during the preparation of food additives based on polyphenols and fibers.

## 1. Introduction

There is increased interest from the industrial and academic sectors in the development of functional foods with bioactive compounds. Among such compounds, polyphenols have received special attention due to various biological activities that promote health. In addition to regulating plant growth and pollination processes, flavonoids are a large subgroup of polyphenols that are responsible for the taste of plants and also serve as colorants. Numerous studies have been performed to prove the various biological activities of flavonoids, which includes anti-inflammatory, antimicrobial, antioxidant, and antitumor action [1,2]. Quercetin belongs to the flavonol subclass of flavonoids and is commonly found in vegetables and fruits such as kale, berries, apples, onions, broccoli, cherries, red grapes, as well as in red wine, and tea [1]. Novel studies have pointed out its role in preventing health issues such as osteoporosis, cardiovascular diseases, and some forms of cancers [3,4,5]. Quercetin exhibits strong antioxidant activity through its effect on the enzymatic system, reactive oxygen species, and signal transduction pathways [6]. Disadvantages such as its hydrophobic nature and poor water solubility limit quercetin’s wider application. New approaches to the development of quercetin carriers and appropriate delivery systems are being applied to overcome its poor solubility in water and enable the biological activity of quercetin in humans [7]. Dietary fibers are recognized as safe and efficient materials for the development of systems with incorporated polyphenols. Fibers, resistant to digestion and absorption in the human small intestine, can carry polyphenols to the lower parts of the digestive tract where they can show positive bioactivities [8]. Many studies have been conducted on polyphenol interactions with dietary fibers and their encapsulation. Polyphenols from blackberry juice were successfully encapsulated onto citrus fiber [9] and apple fiber [10,11]. Vukoja et al. [12] freeze-dried raspberry polyphenols with cellulose and evaluated the stability of such encapsulates. In a study by Ćorković et al. [13], delivery systems of tart cherry polyphenols with carboxymethylcellulose were prepared and characterized. Besides dietary fibers, other polysaccharides (such as starch, maltodextrin, and β-cyclodextrin), lipids, or proteins can be applied as polyphenols carriers. Huang et al. [14] developed liposome–chitosan hydrogel beads to enhance the solubility and stability of quercetin. Kopjar et al. [15] used brown rice and almond protein matrices for the preparation of microparticles with incorporated quercetin. β-glucan was used for the development of complexes with nutraceuticals (quercetin, curcumin, ascorbic acid, coenzyme Q10, boswellic acid, and folic acid). Results indicated the deep penetration of compounds into helices forming the β-glucan matrix and improved the thermal, light, and oxidative stability of bound nutraceuticals of β-glucan [16]. In addition, quercetin was loaded in star-shaped polylactides with β-cyclodextrin, and that formulation exhibited antibacterial properties against *Staphylococcus aureus*, *Escherichia coli,* and *Klebsiella pneumonia* [17]. Another group of authors investigated quercetin inclusion in complex with (2-hydroxypropyl)-β-cyclodextrin and observed the increased solubility of quercetin in water, as well as photostability after encapsulation [18].

A growing area of interest in the food industry is food waste and waste stream utilization. Apple and citrus waste present an inexpensive and valuable material for a wide range of applications. High amounts of dietary fiber present in this waste, mainly pectin, cellulose, and hemicellulose, can be further used for food purposes through appropriate processing [19].

Generally, utilization of plant-based additives in the food industry, agriculture, and the pharmaceutical industry is on the rise, and extensive attention is being devoted to their proper and efficient formulation. Another goal of the food industry is the zero-waste approach; thus, in this study, we used citrus and apple fibers that were classified as waste after production. The aim of this study was to explore the preparation of fiber aggregates as possible plant-based additives. Considering all the above, the present study investigated quercetin behavior in systems with apple and citrus fibers where different concentrations of quercetin solutions (5, 10, and 20 mM) were complexed with fibers. The evaluation of these aggregates was conducted by HPLC (high-performance liquid chromatography) analysis to determine the concentration of quercetin, and spectrophotometric methods (ABTS, DPPH, FRAP, and CUPRAC assays) were used to assess the antioxidant capacity of such aggregates. FTIR-ATR (Fourier transform infrared with attenuated total reflection) analysis was applied to prove that quercetin–fibers interaction occurred, and DSC (differential scanning calorimetry) was used for evaluation of the thermal stability of fiber aggregates.

## 2. Results

Trends in the food industry are concentrated on the utilization of plant-based additives, which can be beneficial in terms of the production of functional food products but also in terms of improving food product quality. With this aim, we formulated quercetin/fiber aggregates based on citrus and apple fibers, which are both valuable by-products in the food industry.

### 2.1. Quercetin Concentrations and Antioxidant Activity of Quercetin/Fiber Aggregates

The concentrations of quercetin present in the aggregates are given in Table 1. It was observed that aggregates with apple fibers had a higher concentration of quercetin, which ranged from 37.76 μg/100 g to 72.01 μg/100 g. Quercetin concentrations in aggregates with citrus fibers ranged from 23.32 μg/100 g to 42.58 μg/100 g. Additionally, results showed that higher amounts of quercetin in the initial solution resulted in higher concentrations in aggregates, but a proportional increase in the concentration of quercetin was not observed. Twice higher concentration of quercetin in the initial solution did not result in twice higher concentration of quercetin in aggregates, regardless of fiber type. When the concentration of quercetin was two times higher in the initial solution, concentrations of quercetin in fiber aggregates were 1.48 and 1.42 times higher for citrus and apple fiber, respectively. On the other hand, when the concentration of quercetin was four times higher in the initial solution, concentrations of quercetin in fiber aggregates were only 1.83 and 1.91 times higher for citrus and apple fiber, respectively.

The antioxidant activity of obtained aggregates is presented in Table 2. Aggregates with apple fiber and quercetin had higher antioxidant activity in all assays. The ABTS assay results ranged from 12.40 μmol/100 g to 23.53 μmol/100 g for citrus fiber aggregates and from 42.57 μmol/100 g to 72.10 μmol/100 g for apple fiber aggregates. The values obtained in the results follow the concentrations of quercetin present in the aggregates. Aggregates with higher quercetin concentrations possessed higher antioxidant activity. The results of DPPH assays ranged from 9.76 μmol/100 g to 14.84 μmol/100 g and from 43.84 μmol/100 g to 55.26 μmol/100 g for citrus and apple fibers aggregates, respectively. The results from FRAP and CUPRAC assays for citrus fibers aggregates ranged from 3.21 μmol/100 g to 4.04 μmol/100 g and from 14.65 μmol/g to 21.23 μmol/g, respectively. By observing FRAP and CUPRAC results for apple fiber aggregates, the same trend as in other assays was noticed, and results ranged from 7.67 μmol/100 g to 13.54 μmol/100 g and from 43.13 μmol/g to 66.11 μmol/g, respectively.

### 2.2. Thermal Stability of Quercetin/Fiber Aggregates

DSC scanning of fiber aggregates was conducted in order to evaluate the thermal stability of aggregates upon adsorption of quercetin. Results for fiber aggregates were compared and the results are presented in Table 3. Apple fibers had a higher melting temperature (90.03 °C) than citrus fibers (85 °C). With the increased concentration of quercetin, changes in this parameter were observed in the aggregates. The behavior of selected fibers was different. For citrus fiber aggregates, it was determined that with increases in quercetin concentration, increases in melting temperature occurred. Apple fibers had the opposite trend; with increases in quercetin concentration, decreases in melting temperature were observed.

### 2.3. FTIR-ATR Spectra of Quercetin/Fiber Aggregates

**Figure 1:**
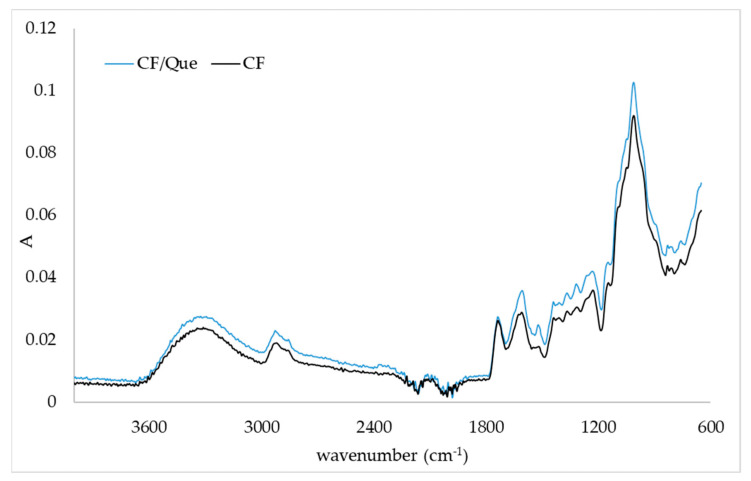
IR spectra of citrus fiber (CF) and citrus fiber/quercetin aggregates (CF/Que).

**Figure 2:**
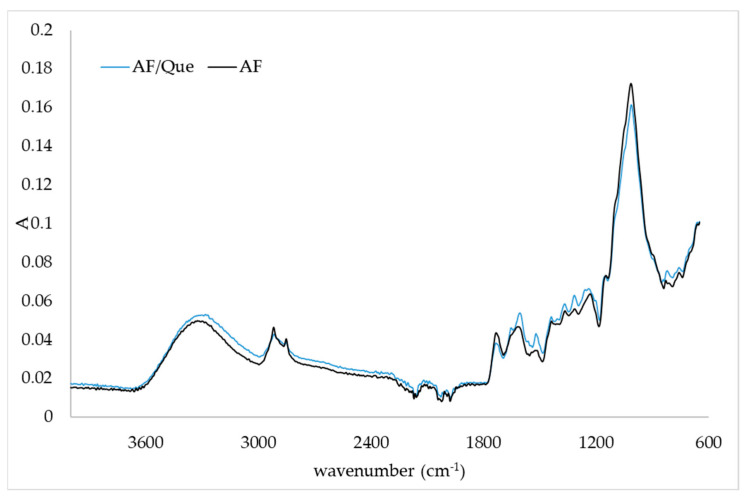
IR spectra of apple fiber (AF) and apple fiber/quercetin complex (AF/Que).

FTIR-ATR analysis was used to observe the main functional groups present in citrus and apple fibers and changes in IR spectrum in the presence of quercetin. Changes in IR spectra in aggregates prepared with different initial concentrations of quercetin were the same, so only one IR spectrum of aggregates is presented. Figure 1 and Figure 2 represent the IR spectra of the citrus fiber and citrus fiber/quercetin aggregates and the apple fiber and apple fiber/quercetin aggregates, respectively. By comparing the IR spectrum of pure fiber and aggregate, changes in fiber structure after the adsorption of quercetin were noticed. By observing Figure 1 and Figure 2, the characteristic broad adsorption band at 3500–3000 cm^−1^ corresponding to the stretching vibration of the hydroxyl group can be noticed. Additionally, bands at 2919 cm^−1^ and 2851 cm^−1^ are assigned to the asymmetric stretching of CH_2_ and refer to the typical structure of polysaccharides [20,21]. The band at 1730 cm^−1^ can be assigned to the C=O stretching vibration of alkyl ester in pectin, and the band at 1600 cm^−1^ can be assigned to COO^-^ antisymmetric stretching on polygalacturonic acid carboxylate (pectin ester group) [22]. The bands at 1428 cm^−1^, 1360 cm^−1^, and 1312 cm^−1^ correspond to CH_2_ symmetric bending on cellulose. The band at 1144 cm^−1^ can be assigned to O-C-O asymmetric stretching on pectin, while the band at 1010 cm^−1^ corresponds to C-C and C-O stretching [22]. Changes in the IR spectrum (Figure 1) of citrus fiber caused by quercetin incorporation are demonstrated by decreased IR spectrum intensity in the regions from 3500 cm^−1^ to 2500 cm^−1^ and 1650 cm^−1^ to 600 cm^−1^. A weak band on the fiber at 1625 cm^−1^ disappeared when quercetin was present. Furthermore, a shoulder at 1565 cm^−1^ in the IR spectrum of citrus fiber/quercetin aggregate was observed. From the IR spectra of the apple fiber and apple fiber/quercetin aggregate (Figure 2), it can be noticed that changes in apple fiber structure occurred when quercetin was present. The first observed change, in the presence of quercetin, was the formation of a band at 1990 cm^−1^. Further, at 1651 cm^−1^, 1252 cm^−1^, and 1200 cm^−1^ on the IR spectrum, complex new bands could be observed. A shift from 1230 cm^−1^ on fiber to 1237 cm^−1^ on fiber/quercetin aggregate also occurred. Additionally, a shift from 820 cm^−1^ on fiber to 842 cm^−1^ on fiber/quercetin aggregate was noticed, and a new band at 816 cm^−1^ on fiber/quercetin aggregate was observed.

## 3. Discussion

Production of functional food based on polyphenols and fibers demands knowledge of the binding nature and interactions between these compounds [23]. Different polyphenol binding selectivity toward different types of polysaccharides was observed. Liu et al. [24] investigated the adsorption of epicatechin, chlorogenic acid, and phlorizin in apple cell walls. They found that in conditions with low concentrations of tested polyphenols, adsorption was concentration-dependent, while in conditions with high concentrations of polyphenols, the number of unoccupied bonds on the cell walls governed adsorption. Other authors have also suggested concentration-dependent adsorption between polyphenols and dietary fibers [25,26]. This study also proved that binding depended on quercetin concentration, and it can be assumed that quercetin adsorption was dependent on the amount of available binding sites on fibers. Additionally, we cannot neglect the higher adsorption of quercetin with apple fibers than with citrus ones. This could be assigned to the different compositions of used fibers, which caused different binding capacity for quercetin. According to our results, the higher affinity of quercetin toward apple fibers can be related to the water adsorption capacity (WAC) of investigated fibers. We evaluated that WAC was 9.46 g for citrus fibers and 4.23 g for apple fibers, which proved that apple fiber is more hydrophobic than citrus fiber. Quercetin is also a hydrophobic compound, with a log *P* value of 1.82 [27]. Previous studies have shown the impact of cell wall composition on polyphenol adsorption. In the case of procyanidin adsorption, a higher affinity has been shown toward citrus pectin than apple pectin [28]. These results were opposite to ours, which could indicate that the type of polyphenol, as well as fiber composition and properties, affected adsorption.

Antioxidant activity related to a positive impact on various diseases was correlated to quercetin and has been well investigated [29]. To confirm that our samples possess such properties, four different antioxidant assays (ABTS, DPPH, FRAP and CUPRAC) were conducted. The advantage of using more than a single method is that the overall antioxidant profile of tested materials can be obtained due to the different mechanisms of the applied methods. In the food industry, assays based on the reaction of ABTS^•+^ and DPPH^•^ radicals with antioxidants are the favored spectrophotometric methods for the evaluation of antioxidant activity [30]. These methods are suitable due to their simple, highly sensitive, reproducible, and inexpensive procedures. ABTS radicals are more reactive than DPPH radicals. CUPRAC and FRAP assays are based on the interactions of copper and iron metals with antioxidants in the sample [30,31]. From the results of antioxidant activities, evaluated by ABTS and DPPH assays, it can be observed that citrus fiber aggregates possessed a higher capability to reduce ABTS radicals. Results of FRAP and CUPRAC assays pointed out that all examined samples had higher reducing capability for Cu(II) to Cu(I) than for Fe(III) to Fe(II). Similar results were observed in a study by Kopjar et al. [15], where quercetin was adsorbed onto brown rice and almond protein matrices. They evaluated the antioxidant activity of obtained microparticles and observed the same trend regarding reduced capability in CUPRAC and FRAP assays, as also indicated by our results. Studies have shown that the antioxidant activity of quercetin is higher when it is complexed with other compounds, such as metal ions [32,33], or in glucan–quercetin conjugates and quercetin–calcium phosphate nanocomplexes [34]. Zou et al. [35] encapsulated quercetin in biopolymer-coated zein nanoparticles, and their results for antioxidant activity (evaluated by ABTS^•+^ scavenging capacity and an Fe^3+^ reducing power assay) revealed better antioxidant properties when quercetin was encapsulated.

Structural changes in fibers caused by polyphenol adsorption can be proven using FTIR-ATR analysis. Moon et al. [29] encapsulated quercetin into soybean polysaccharide/chitosan and noticed small changes in the IR spectrum (some weak bands appeared or disappeared in the presence of quercetin). They suggested hydrophobic interactions as driving forces for the encapsulation of quercetin. In a study by Savic et al. [18], where quercetin was encapsulated in (2-hydroxypropyl)-β-cyclodextrin, the bands of the carrier were more dominant in the spectrum than quercetin bands. This is in accordance with our results where only small changes were observed in the presence of quercetin. Hao et al. [1] and Veverka et al. [16] suggested hydrophobic interactions (hydrogen bonds and van der Waals forces) between quercetin and encapsulated material, which could also have been a driving force for the interactions between quercetin and citrus or apple fibers in this study.

As in other studies [36,37,38], DSC was the tool that was used for evaluation of the thermal stability of fibers and fiber aggregates. The addition of young apple polyphenols for the enrichment of citrus pectin films led to the enhanced thermal stability of formulated films [36]. On the other hand, the thermal stability of the chitosan films, determined by DSC, decreased with addition of the same type of polyphenols [38]. In both studies, structural changes that were induced by the binding of polyphenols on polymers were underlined as the reason for the change in the thermal stability of formulated films. Additionally, structural changes were proven by FTIR-ATR analysis [36,38]. The thermal behavior of pectin molecules is largely defined by their internal and external bonding and their configuration [39], and these factors can be translated to our samples since both fibers used for this study contained pectin. Changes in the degradation temperature of fiber aggregates can be linked to hydrogen bonds formed between quercetin and fiber constituents (pectin, cellulose). In this study, we also proved that there are structural changes in fiber aggregates upon the binding of quercetin on citrus and apple fibers. However, in the case of citrus fiber, these changes caused an improvement in thermal stability, while the reverse effect was observed in apple fiber.

## 4. Materials and Methods

### 4.1. Materials

Apple fibers were obtained from Biesterfeld AG (Zagreb, Croatia) and citrus fibers were obtained from Fiberstar (River Falls, WI, USA). Gallic acid, 2,2′-azino-bis(3-ethylbenzothiazoline-6-sulfonic acid) diammonium salt (ABTS), trolox, 2,2-diphenyl-1-picrylhydrazyl (DPPH), and quercetin were obtained from Sigma-Aldrich (St. Louis, MO, USA). Neocuproine, 2,4,6-tri(2-pyridyl)-s-triazine (TPTZ), and copper (II) chloride were purchased from Acros Organics (Geel, Belgium). Methanol (HPLC grade) was obtained from J.T. Baker (Deventer, Netherlands) and orthophosphoric acid (HPLC grade > 85%) was obtained from Fisher Scientific (Loughborough, UK). Iron (III) chloride hexahydrate, sodium acetate, ethanol, and ammonium acetate were purchased from Grammol (Zagreb, Croatia).

### 4.2. Preparation of Quercetin/Fiber Aggregates

For the creation of quercetin solutions (5 mM, 10 mM, and 20 mM), 96% ethanol was used. In a 50 mL quercetin solution, 1% of apple or citrus fiber was added. Complexation was performed for 30 min at room temperature using a magnetic stirrer (Stuart US152, Buch and Holm, Hervel, Denmark). Afterward, mixtures were centrifuged for 15 min at 4000 rpm. The supernatant was then discarded, and the precipitate was air-dried overnight.

### 4.3. Extraction of Quercetin/Fiber Aggregates

Around 0.2 g of the aggregate was weighed and mixed with 5 mL of acidified methanol (methanol: HCl ratio was 99:1 (*v/v*)). Ultrasound-assisted extraction was performed for 20 min and mixtures were then centrifuged for 20 min at 4000 rpm. The supernatant was used for both the evaluation of quercetin concentration via HPLC analysis and the evaluation of antioxidant activity through ABTS, DPPH, FRAP, and CUPRAC assays.

### 4.4. High-Performance Liquid Chromatography (HPLC) Analysis of Quercetin Concentrations

The Agilent HPLC system 1260 Infinity II (Agilent Technology, Santa Clara, CA, USA) equipped with a diode array detector (DAD), a quaternary pump, a vial sampler, and a Poroshell 120 EC C-18 column (4.6 × 100 mm, 2.7 µm) was used for determination of quercetin concentrations in complexes. Prior to injection, 0.5 mL of extract was filtered through a 0.45 µm PTFE syringe filter. The chromatographic separation was performed with 0.1% orthophosphoric acid as mobile phase A and 100% methanol as mobile phase B with the following gradient: 0 min, 5% B; 3 min, 30% B; 15 min, 35% B; 22 min, 37% B; 30 min, 41% B; 32 min, 45% B; 40 min, 49% B; 45 min, 80% B; 48 min, 80% B; 50 min, 5% B; 53 min, 5% B. Flow rate was 1 mL/min and the injection volume was 10 µL. A stock solution of quercetin was prepared in 100% methanol and used for the construction of the quercetin calibration curve, with concentrations ranging from 5 mg/L to 150 mg/L (r^2^ = 0.9998; LOD = 0.602 mg/L; LOQ = 1.82 mg/L). Areas of peaks were read at 360 nm. Measurements were conducted twice.

### 4.5. Evaluation of the Antioxidant Activity of Quercetin/Fiber Aggregates

The antioxidant activity of the prepared aggregates was evaluated by ABTS, DPPH, FRAP, and CUPRAC assays. The ABTS assay was carried out according to Arnao et al. [40] with slight modifications. Obtained extracts were mixed with 3.2 mL of ABTS reagent and incubated for 95 min in the dark, and absorbance was measured at 734 nm using a UV/Vis spectrophotometer (Cary 60 UV-Vis, Agilent Technologies, Santa Clara, CA, USA). The protocol of the DPPH assay was that described by Brand-Williams et al. [41]. Briefly, extracts were mixed with 3 mL of DPPH solution (0.5 mM). The mixture was then incubated for 15 min in the dark and absorbance was read at 517 nm. Apak et al. [31] described the protocol for the CUPRAC assay used in this study. Briefly, 10 mM CuCl_2_ (1 mL), 7.5 mM neocuproine (1 mL), 1M ammonium acetate buffer pH 7.0 (1 mL), extract, and distilled water (total volume of 1.1 mL) were mixed and incubated for 30 min in the dark. The absorbance was measured at 450 nm. The FRAP assay was performed according to Benzie and Strain [42]. Extracts and FRAP reagent were incubated for 30 min in the dark and measured at 593 nm. All assays were performed in triplicate and the results were expressed as micromoles of Trolox equivalent per 100 g of sample (µmol TE/100 g).

### 4.6. Determination of the Water Adsorption Capacity of Fibers

The water adsorption capacity of fibers was determined according to Wang et al. [43] with slight modifications. Briefly, 1 g of fiber was mixed with 50 mL of water in the magnetic stirrer. After 15 min of mixing, the obtained mixtures were centrifuged for 10 min at 1000× *g*. Supernatants were discarded, and the precipitates were weighed. Measurements were conducted twice.

### 4.7. Differential Scanning Calorimetry (DSC) of Quercetin/Fiber Aggregates

The DSC screening of fibers and aggregates was conducted on a differential scanning calorimeter (Mettler Toledo 822, Mettler Toledo, Greifensee, Switzerland). Firstly, 5 mg of sample was weighed in a 40 μL aluminum pan, which was then covered and inserted into the DSC oven. Screening of samples was performed from 25 °C to 120 °C. At the beginning of the screening, samples were left for 4 min at 25 °C. Afterward, the temperature was increased at a rate of 10 °C/min up to 120 °C, where samples were also left for 4 min. Screenings were performed twice.

### 4.8. FTIR-ATR (Fourier Transform Infrared with Attenuated Total Reflection) Analysis of Quercetin/Fiber Aggregates

A Cary 630 system (Agilent, Santa Clara, CA, USA) equipped with MicroLab Expert software was used to record the infrared spectra (IR) (from 4000 cm^−1^ to 600 cm^−1^) of the obtained complexes.

### 4.9. Statistical Analysis of Obtained Results

The STATISTICA 13.1 (StatSoft Inc., Tulsa, OK, USA) software program was utilized to analyze the obtained results. Variance analysis (ANOVA) and Fisher’s least significant difference (LSD) test, with significance defined as *p* < 0.05, were selected for statistical evaluation of the results, which are presented as mean value ± standard deviation.

## 5. Conclusions

Three different initial solutions of quercetin (5 mM, 10 mM, and 20 mM) were subjected to complexation with two different types of fibers: citrus and apple fibers (which are valuable by-products of the food industry). Obtained results show that quercetin had higher adsorption capability toward apple fibers than citrus fibers. In addition, it was observed that quercetin adsorption was not proportional to quercetin concentration and depended on the number of available binding sites on fibers. These results and findings might be of use during the formulation of food additives or nutraceutical formulations based on flavonoids and dietary fibers, especially since the utilization of plant-based food additives is on the rise. Future studies need to examine the behavior of such fiber aggregates in real food systems.

## Figures and Tables

**Table 1:** Quercetin concentrations in fiber aggregates (μg/100 g).

Que_5	Que_10	Que_20
Citrus fiber aggregates		
23.32 ± 0.31 ^c^	34.49 ± 0.03 ^b^	42.58 ± 0.15 ^a^
Apple fiber aggregates		
37.76 ± 0.37 ^c^	53.66 ± 0.19 ^b^	72.01 ± 0.64 ^a^

Que, quercetin; 5, 10, and 20 represent 5 mM, 10 mM, and 20 mM initial quercetin solutions. Results are presented as mean value ± standard deviation of two replicates. Within the row (separately for citrus fiber/quercetin and apple fiber/quercetin aggregates), means followed by different superscript letters are significantly different at *p* ≤ 0.05 (ANOVA, Fisher’s LSD).

**Table 2:** Antioxidant activity of fiber aggregates.

Samples	ABTS (μmol/100 g)	DPPH (μmol/100 g)	FRAP (μmol/100 g)	CUPRAC (μmol/g)
Citrus fiber aggregates				
Que_5	12.40 ± 0.10 ^c^	9.76 ± 0.43 ^c^	3.21 ± 0.15 ^b^	14.65 ± 0.80 ^b^
Que_10	18.09 ± 0.18 ^b^	12.51 ± 0.30 ^b^	3.87 ± 0.20 ^a^	19.58 ± 1.63 ^a^
Que_20	23.53 ± 0.08 ^a^	14.84 ± 0.52 ^a^	4.04 ± 0.22 ^a^	21.23 ± 0.79 ^a^
Apple fiber aggregates				
Que_5	42.57 ± 0.47 ^c^	43.84 ± 1.33 ^c^	7.67 ± 0.28 ^c^	43.13 ± 1.34 ^c^
Que_10	48.15 ± 0.94 ^b^	47.77 ± 0.20 ^b^	9.02 ± 0.20 ^b^	51.98 ± 1.13 ^b^
Que_20	72.10 ± 0.82 ^a^	55.26 ± 0.95 ^a^	13.54 ± 0.17 ^a^	66.11 ± 1.30 ^a^

Que, quercetin; 5 mM, 10 mM, and 20 mM initial quercetin solutions. Results are presented as mean value ± standard deviation of three replicates. Within the column (separately for citrus fiber/quercetin and apple fiber/quercetin aggregates), means followed by different superscript letters are significantly different at *p* ≤ 0.05 (ANOVA, Fisher’s LSD).

**Table 3:** Melting temperatures of fibers and quercetin/fiber aggregates (°C).

CF	85.00 ± 0.01 ^d^	AF	90.03 ± 0.02 ^a^
CF/Que_5	85.44 ± 0.09 ^c^	AF/Que_5	89.29 ± 0.07 ^b^
CF/Que_10	85.95 ± 0.08 ^b^	AF/Que_10	88.03 ± 0.09 ^c^
CF/Que_20	89.58 ± 0.07 ^a^	AF/Que_20	86.23 ± 0.09 ^d^

CF, citrus fiber; AF, apple fiber; Que, quercetin; 5, 10, and 20 represent 5 mM, 10 mM, and 20 mM initial quercetin solutions. Results are presented as mean value ± standard deviation of two replicates. Within the row (separately for citrus fiber/quercetin and apple fiber/quercetin aggregates), means followed by different superscript letters are significantly different at *p* ≤ 0.05 (ANOVA, Fisher’s LSD).

## Data Availability

Not applicable.
